# Emerging Pediatric HIV Epidemic Related to Migration

**DOI:** 10.3201/eid1204.051025

**Published:** 2006-04

**Authors:** Douglas W. MacPherson, Militza Zencovich, Brian D. Gushulak

**Affiliations:** * Migration Health Consultants Inc., Cheltenham, Ontario, Canada;; † McMaster University, Hamilton, Ontario, Canada;; ‡ Citizenship and Immigration Canada, Ottawa, Ontario, Canada

**Keywords:** pediatric, HIV/AIDS, immigrant, medical screening, treatment, health policy

## Abstract

In 2002, Canada introduced routine, mandatory HIV antibody screening for all residency applicants, including selected children. We report screening results from January 2002 to February 2005. Thirty-six pediatric HIV cases were detected (14/100,000 applicants); 94% of infected children were eligible to arrive in Canada. Thirty-two of the affected children were from Africa, and maternal infection was the main risk factor. Only 4 (11%) of the children had received antiretroviral therapy. In countries of low HIV incidence, migration-related imported infection in children may be an emerging epidemic. The early identification of HIV-infected immigrant women permits intervention to prevent mother-to-child HIV transmission. Routine HIV testing as a component of the medical examination of immigrants has national and international health policy and programmatic implications.

## Abstract 2

Imported HIV infection is an emerging epidemic in countries with low HIV incidence.

In 2004, the Joint United Nations Programme on HIV/AIDS reported that the number of persons living with HIV and AIDS in the world was 39.4 million, 4.9 million were newly infected with HIV, and 3.1 million had died because of HIV/AIDS (*1*). Within these numbers are an estimated 2.2 million children <16 years of age who are living with HIV/AIDS. Globally in 2003, ≈640,000 new HIV pediatric infections and 510,000 pediatric deaths occurred. Most pediatric HIV/AIDS occurs in the developing world because of mother-to-child transmission, but pediatric HIV/AIDS is also a concern in Western nations, where several strategies have been developed and implemented to prevent pediatric HIV infection and subsequent AIDS-related disease.

A link between HIV infection in hyperendemic zones of the developing world and pediatric HIV infections in Western countries is largely undocumented (*2*). Irregular migrants (those who arrive by smuggling or trafficking) and those seeking asylum in Europe represent a separate epidemiologic pattern of HIV/AIDS introduction (*3*). For regions without extensive immigration assessment programs, or where most international arrivals for permanent residency are seeking asylum or are arriving through other irregular means, migration-associated HIV/AIDS prevalence and the pediatric HIV/AIDS epidemic are emerging policy and programmatic issues. Injection drug use and sexual exploitation, particularly for women, are 2 potential risks associated with illegal immigration status that increase HIV exposure, with the potential consequence of mother-to-child viral transmission (*4*).

Prearrival immigration medical screening has been used to identify conditions such as tuberculosis, syphilis, and HIV/AIDS that could affect admission to receiving nations. Many immigrant-receiving nations in the industrialized world now have national policies designed to better address the needs of vulnerable, foreign-born migrants or to facilitate the immigration process for preferred applicants. Nations who either have existing medical screening programs or who are planning such programs are likely to identify persons with HIV/AIDS. Decisions on screening immigrants for HIV infection not only have direct implications for admissibility programs but also affect the need for culturally and linguistically appropriate clinical and public health services.

The 2002–2003 annual report of the Ministerial Council on HIV/AIDS in Canada "estimated that 70% of all maternal HIV transmissions to children in Canada have occurred among women of African and Caribbean origin" (*5*). From November 1985 to June 2004, the Public Health Agency of Canada reported notification of 56,523 positive HIV test results (*6*). From 1984 to 2002, it also reported 420 HIV infections in 1,584 children born to HIV-positive mothers (*7*).

Apart from the potential for perinatal HIV exposure, other pediatric risk factors for nonmaternal HIV acquisition in the industrialized world, such as blood transfusion, tattooing, or illicit drug use, are rarely encountered or documented. Strategies designed to reduce mother-to-child HIV transmission and pediatric HIV infection include HIV screening programs for pregnant women (*8*), risk behavior counseling, recommendations for antiretroviral treatment to prevent mother-to-child-transmission or to treat a newborn exposed to HIV at birth, conception control, and birthing methods. These strategies have been relatively successful in reducing pediatric HIV infections in most industrialized nations but have been less effective in developing nations (*9**–**11*).

The role of population mobility between hyperendemic HIV transmission zones and countries of lower prevalence is emerging as a contributing factor in risk for pediatric HIV infection between HIV high-prevalence and low-prevalence regions. This article describes the results of the first 3 years of a medical screening program for HIV antibodies in selected children who were applicants for residency in Canada. The results of this study confirm that mobile population dynamics between countries and regions and demographic changes in pediatric HIV/AIDS epidemiology are directly related and have consequences for immigration and health policy at domestic and international levels.

## Methods

### Population

Mandatory, routine serologic HIV testing of immigrants in Canada began in January 2002 as a component of the Canadian immigration medical examination (*12**,**13*). While routine testing was not conducted on applicants <15 years of age, those identified as being at risk in the pediatric migrant populations were evaluated for HIV infection. An enzyme-linked immunoassay (EIA) HIV screening test for HIV-1 and HIV-2 antibodies was required for all children <15 years of age who had received blood or blood products, had an HIV-positive mother, or in the judgment of the assessing medical practitioner, was noted to have any other identified HIV infection risk factor. Potential adoptees were also screened for HIV antibodies until a policy decision changed this requirement in late 2004. Reflecting national and international legal processes of adoption, international adoptees brought to Canada by Canadian citizens or permanent residents of Canada are considered part of the existing family, and although they are required to apply for Canadian citizenship, they are exempted from most immigration processes (including the medical examination to determine inadmissibility).

The HIV-tested migrant groups included children of applicants for permanent residence (immigrants and refugees) and those who filed refugee or asylum claims in Canada. Immigration medical screening, including routine HIV testing for those >15 years of age is also required for some other persons arriving in Canada, including visitors staying >6 months from certain locations (e.g., tourists, students, and seasonal workers). Children of persons in this group could also be referred for HIV testing if risk factors were noted during the process (*14*).

### Medical Assessment and HIV Testing Protocols

The guardians of all pediatric applicants were counseled and consented to HIV testing. Venous blood was collected and transported to an approved local testing facility. HIV antibody was tested by EIA. Immunofluorescence antibody testing and Western blot (or other approved manufacturer's EIA kit) testing on another blood sample were used as confirmatory tests on positive or indeterminate HIV antibody test results.

### Data Management and Protection of Personal Information

Immigration medical screening data were provided by Citizenship and Immigration Canada. Personal data were protected according to national guidelines on information privacy (*15**,**16*). Age was calculated from the date of birth and application date on the immigration file. The 5 immigration categories used in the analysis, reflecting current Canadian classifications, were economic, family, refugee (abroad), refugee claimant (in Canada), and other (*17*). The "other" category includes temporary resident applicants, such as visitors, workers, and students.

Currently, Canadian immigration policy reflects 3 major goals. New immigrants are selected on the basis of 1 of these 3 basic principles: reunification of families, economic ability to become successfully established in Canada, or humanitarian reasons based on displacement or persecution. Specific components of each of these major immigration selection groups are further described by legislation and regulation (*18*).

Refugee claimants, known as asylum seekers in Europe, differ from refugees whose convention-defined status has been determined before arrival (*19**–**21*). Refugee claimants arrive through uncontrolled and irregular means and complete the immigration medical assessment processes entirely within Canada as part of the procedures for determining their refugee status. During the past decade, the number of refugee claimants arriving in Canada has varied from 23,000 to 43,000 claimants per year (*22*). Since the implementation of the Safe Third Country Agreement between Canada and the United States on December 29, 2004, the number of refugee claimants has declined by ≈25% (*23*).

## Results

From January 2002 to February 2005, a total of 1,307,718 persons underwent a Canadian immigration medical assessment (*24*), including 256,970 applicants (124,195 female, 132,775 male) <15 years of age. Thirty-six new HIV-positive diagnoses were made in the pediatric applicant population; 18 were boys and 18 were girls. Twelve HIV-positive applicants were <1 year of age at diagnosis, 7 were 2–4 years, 7 were 5–7 years, 2 were 8–10 years, and 8 were 11–14 years. Median age for girls was 6 years (range from infancy to 14 years) and for boys, 4 years (range from infancy to 12 years). All HIV-infected children except 4 (2 from Europe, 2 from Asia/Pacific Islands) identified a country in Africa as either their parents' home or their country of birth. Twenty-seven (75%) of the 36 HIV-infected children were tested because of known maternal HIV positivity, 6 children (17%) were tested because of adoption, 2 (6%) were tested because of an HIV-positive sibling, and 1 (3%) was detected because of concurrent treatment for tuberculosis. Two girls (ages 6 and 12 years) and 2 boys (ages 6 and 11 years) (11%) had documentation of having received highly active antiretroviral therapy (HAART). Two of the 4 received HAART in North America, 1 in the United Kingdom, and 1 in Ethiopia. By applicant category, 2 of the children receiving HAART were family class, 1 was a refugee claimant, and 1 was an economic applicant.

HIV-positive children by immigration category and year of testing are shown in the Table. Refugees represented 26 (72%) of the 36 HIV diagnoses, family class represented 7 (19%), and economic, refugee claimant, and temporary resident applicants represented 1 case each (3%). Only 2 HIV-infected children were processed in the immigration medical office responsible for North America, which indicates their application originated in Canada or the United States.

**Table:** Immigration applicants and HIV-positive pediatric applicants by immigration category and year

Immigration applicants	2002	2003	2004	2005*	Total	% by category
Total applicants	347,438	430,259	455,553	74,468	1,307,718	
Total applicants <15 years of age	68,734	81,540	92,055	14,641	256,970	
Immigration category of HIV-positive pediatric applicants						
Economic	0	1	0	0	1	3
Family class	1	4	1	1	7	19
Refugees	3	6	12	5	26	72
Refugee claimants	1	0	0	0	1	3
Other (temporary residents)	0	1	0	0	1	3
Total	5	12	13	6	36	100

*January and February 2005 only.

Thirty-three (92%) of the 36 HIV pediatric cases occurred in populations deemed to be eligible for admission to Canada, despite medical status, on the basis of these application categories (26 in the refugee class and 7 in the family class). Of those children found to be HIV positive in the study period, 24 (66%) had arrived in Canada.

## Discussion

In the context of all immigration applicants, 36 HIV-infected children were identified. During the study, 256,970 applicants <15 years of age underwent medical examinations. This number represents a crude ratio of 14/100,000 pediatric applicants from January 2002 through February 2005. A ratio per tested pediatric applicant cannot be determined because negative serologic test results are not recorded for this age group, and no estimate of the tested population size is possible because of variations in medical examiner and adjudicator practices during the study. All but 4 of the pediatric HIV -positive applicants were originally from Africa (89%), which reflects the relationship between population flow and the global epidemiologic features of HIV infection and disease (*25*). In contrast, domestically reported pediatric HIV cases are rare in Canada (0.02/100,000 general population).

Applicants in the refugee (26 cases) and family (7 cases) categories accounted for 33 (91%) of the 36 HIV diagnoses in this study period. The refugee and family categories are exempted from provisions that can render applicants inadmissible because of medical reasons. Consequently, the 33 children in these groups were all medically eligible to be admitted to Canada. When those children are combined with the HIV-positive pediatric refugee claimant who was in Canada when tested, 34 (94%) of the 36 HIV-infected children detected during the first 3 years of the mandatory immigration screening program were medically admissible or had already arrived in Canada.

In the 2004 World Health Organization report, Canadian public health officials estimated an overall HIV prevalence ratio in Canada of 3–5 cases per 10,000 pregnant women on the basis of pregnancy HIV screening results (*26*). Fecundity data in Canada from 1986 to 2001 show that 1,197,300 children were born to Canadian-born women and 337,700 children were born to immigrant women (1,535,000 total newborns) (*27*). If the estimated HIV prevalence rate for pregnant women in Canada is applied to the average of 102,333 newborn children per year, an estimated 92–154 singleton births occur to at-risk pregnant women.

The actual number of documented mother-to-child HIV exposures reported in Canada has been less than that figure. From 1984 to 2002, national statistics in Canada show that 1,584 infants (an average of 88 per year) were perinatally exposed to HIV. Of these infants, 420 have been reported as HIV infected. An additional 120 children had unconfirmed HIV status, including those with indeterminate serologic status, those who died, or those who were lost to follow-up. As a possible reflection of the effectiveness of the national perinatal HIV screening, mother-to-child transmission–prevention programs, and other factors, <4% of at-risk pregnancies in HIV-positive mothers resulted in viral transmission to their newborns in 2001 and 2002, with only 12 pediatric HIV cases reported in those 2 years (0.02 cases/100,000 population per year).

As indicated in this study of immigrant applicants, more HIV-infected children are detected through selective immigration medical screening in migrant pediatric populations arriving or arrived in Canada. Growing global population mobility and immigration could more than double the annual domestic pediatric HIV/AIDS caseload in Canada.

Despite the success of industrialized nations' domestic mother-to-child transmission prevention programs, the potential for HIV infection in children still exists in Western society. For several reasons, including education, language, culture, and fear of personal and social reprisals, foreign-born migrant women may have limited access to healthcare services or delay medical care. They may not be able to fully access prenatal care or HIV screening programs that could benefit them and their unborn children (*28**,**29*). Pregnant migrants and migrating women of childbearing potential may be a source for pediatric HIV cases in migrant-receiving nations.

The required medical examination for persons applying for residence from abroad can precede their arrival in Canada by up to 12 months, which raises the possibility of new maternal HIV infection, the new conception of a child, and new birth to HIV-infected mothers. The immigration medical examination and processing represents a time-limited opportunity to detect at-risk pregnancies to provide treatment to prevent mother-to-child transmission of HIV. The medical assessment, including HIV antibody testing, may identify several groups of foreign-born women at risk for transmitting HIV to their children, including women with defined risk factors who are pregnant or may become pregnant during the immigration application process, while in Canada or abroad.

An immigration application provides an opportunity to inform and educate all applicants, particularly women of childbearing potential, of the benefits of HIV screening in pregnancy. Even women who test negative for HIV antibodies during the immigration medical examination should be retested if they become pregnant, whether they are still abroad or have arrived in Canada. The medical examination is also an opportunity to identify resources for HIV screening in pregnancy for these women, separate from the immigration process, and the local access points for maternal management and, if needed, antiretroviral treatment to reduce the risk for perinatal HIV transmission. Three of the 4 children who received HAART in this study did so in Western countries. None of the remaining 32 HIV-infected children, all of whom were in developing nations, had any indication of receiving HAART for either prevention or treatment.

Opportunities for active, programmatic intervention to protect the health of pregnant women and reduce the risk for HIV infection and transmission during pregnancy exist for both refugees and refugee claimants on the basis of their category of application. By definition, refugees have already had their status determined and have already come under the jurisdiction of a national or international authority, such as the United Nations High Commission for Refugees. Programs on health promotion and HIV infection and disease prevention in pregnant women and women of childbearing potential can be part of the international protection offered to this vulnerable population (*30*). In this study, 72% of all pediatric HIV infections were in the refugee category.

By definition, refugee claimants and asylum seekers make their applications from within the host country. In Canada, refugee claimants are provided access to healthcare services while their claim is determined. In spite of the challenges of providing culturally and linguistically accessible healthcare programs for foreign-born, migrant women, this situation offers an opportunity to educate and test pregnant applicants. In this study, one 6-year-old refugee claimant with HIV, who traveled from Africa, received HAART in Canada.

Other risk groups include women known to be HIV positive and their children with risks for exposure to HIV infection, such as breastfeeding; children with risk factors for HIV infection other than birth to an HIV-positive mother; and foreign-born children being adopted. Failure to recognize and use these opportunities may have implications for maternal and pediatric HIV infection in migrant populations.

HIV antibody testing in immigrants is primarily a part of the administrative process of determining the medical status of migrants in accordance with immigration legislation (*31**,**32*). As such, it may not be directly linked to HIV/AIDS clinical management programs. Routine immigration testing for diseases such as HIV/AIDS or tuberculosis provides an opportunity to identify groups at increased risk that may benefit from specific health promotion and disease prevention programs (*33*).

Given the emerging patterns of global HIV/AIDS epidemiology and current immigration patterns, similar situations could be observed over time in other locations. A proportional shift in total and pediatric HIV cases related to foreign-born migrant arrivals in immigrant-receiving nations, where cases in children are less common, can be anticipated. Migrants, and the communities of newly arrived persons that they tend to gravitate to, often have health needs that may differ substantially from those of the host population. The shifting demographics of pediatric HIV infection in Canada can thus be expected to influence local aspects of healthcare planning and delivery. The multicultural aspects of HIV infection in immigrants will affect case management by local public health authorities, social services, clinical pediatric HIV/AIDS services, and other health and social service providers. Existing programs, designed in the context of domestically acquired infections, may not have considered either the size of the population or the culturally and linguistically diverse characteristics of immigrant, HIV-infected children. Other unanticipated program effects, including policy expectations from the international community on the medical, cultural, and social aspects of pediatric HIV/AIDS and immigration should receive greater attention (*34**,**35*).

One of the potential consequences of this shared knowledge related to pediatric HIV and AIDS for domestic program development could be the integration of immigration medical programs and public health programs to prevent mother-to-child transmission of HIV overseas. Only 4 (11%) of 36 of HIV-infected children received specific antiretroviral therapy, and none of the 12 HIV-positive children who were <1 year of age, nor their mothers, were known to have received perinatal HAART to prevent HIV transmission (*36**,**37*). Programs and strategies designed to mitigate some infectious disease risks in migrant populations before arrival have already been used in some situations (*38*); similar rationales could be evaluated for HIV infection in those already involved in immigration formalities.

The analysis and opinions expressed in this article are those of the authors and are not to be attributed to Citizenship and Immigration Canada or the government of Canada, and they do not necessarily reflect or represent the position of any government department, agency, university, or professional society to which the authors may belong or have belonged.
